# Frailty as an Independent Predictor of Mortality in Patients with Sepsis

**DOI:** 10.3390/jpm15090398

**Published:** 2025-08-26

**Authors:** Alejandro Interián, Fernando Ramasco, Angels Figuerola, Rosa Méndez

**Affiliations:** 1 Independent Researcher, Madrid 28006, Spain; 2 Anesthesiology and Surgical Critical Care Department, University Hospital of La Princesa, 28006 Madrid, Spain; fernando.ramasco@salud.madrid.org (F.R.); rmendezh@salud.madrid.org (R.M.); 3 Preventive Medicine and Public Health Department, University Hospital of La Princesa, 28006 Madrid, Spain; angels.figuerola@salud.madrid.org

**Keywords:** sepsis, frailty, comorbidity, mortality, anemia, Intensive Care Unit

## Abstract

**Objectives**: Personalized sepsis care requires understanding how pre-existing health status can influence outcomes. The aim of this study is to evaluate its impact on in-hospital and 12-month mortality in patients with sepsis, taking into account age, comorbidities, the Charlson Comorbidity Index, frailty, anemia, and the Sequential Organ Failure Score Assessment (SOFA) in the first 24 h. **Methods**: An observational and retrospective study was conducted using data from the Sepsis Code program at the Hospital Universitario de La Princesa. The relationship between risk factors and mortality, as well as Intensive Care Unit (ICU) admission, was analyzed for the period 2016–2018 using bivariate and multivariate logistic regression. **Results**: A total of 547 patients were included. In the multivariate analysis, the risk factors independently associated with mortality were Rockwood Clinical Frailty Scale ≥ 5 (OR 2.45, *p* < 0.05); SOFA ≥ 4 (OR 2.13, *p* < 0.05); age (OR 1.98, *p* < 0.05); anemia (OR 1.85, *p* < 0.05); and specific comorbidities such as ischemic heart disease (OR 2.34, *p* < 0.05), severe liver disease (OR 3.62, *p* < 0.05), and metastatic cancer (OR 3.14, *p* < 0.05). Patients who were frail, had dementia, or heart failure were less likely to be admitted to the ICU. **Conclusions**: Frailty, comorbidities, age, and anemia are associated with outcomes in patients with sepsis and could be incorporated into mortality prediction models to guide tailored treatment strategies.

## 1. Introduction

Sepsis is defined as a life-threatening organ dysfunction caused by a dysregulated host response to infection. According to the Sepsis-3 definition, the presence of organ failure—reflected by a Sequential Organ Failure Assessment (SOFA) score ≥ 2—is associated with a mortality rate exceeding 10%, while progression to septic shock may lead to mortality rates greater than 40% [1].

The importance of the early and protocolized identification of sepsis has been emphasized by the Surviving Sepsis Campaign guidelines [2], which recommend the implementation of Sepsis Code-type programs as a key strategy for early detection and systematic management of patients with sepsis. At the Hospital Universitario de La Princesa, the implementation of the Sepsis Code has been associated with improvements in patient care and outcomes [3].

Managing sepsis continues to impose a significant clinical burden and requires intensive use of both human and economic resources, with a direct impact on hospital occupancy, need for advanced support, and healthcare costs [4]. Given that sepsis is a heterogeneous syndrome and host responses vary widely due to patient factors, a “one-size-fits-all” approach may be ineffective [5]. Having tools that can predict which patients are at greater risk of mortality or may require admission to the Intensive Care Unit (ICU) is essential for optimizing resource allocation.

Among the tools used in the initial management of sepsis, the SOFA score stands out [6]. This scale has proven useful for diagnosing sepsis, predicting in-hospital mortality, and anticipating the need for intensive support [1], establishing it as a cornerstone in the stratification of septic patients.

Although SOFA quantifies the severity of organ failure, it does not account for the patient’s baseline health status. The SOFA score’s exclusive focus on acute organ dysfunction represents a critical gap in personalized risk assessment. Combining it with pre-existing health variables could provide a more comprehensive and individualized risk assessment, enabling optimized resource allocation and therapeutic management [7].

Recent studies examining the relationship between pre-existing health status and sepsis outcomes report conflicting results [8,9]. While some indicate higher mortality in previously healthy patients, others emphasize the predominant role of preexisting comorbidities in adverse outcomes. These conflicting results underscore the heterogeneity within the sepsis patient population and reinforce the need for a personalized and multifactorial assessment.

The Charlson Comorbidity Index (CCI) [10] is a widely used tool to quantify the burden of chronic diseases and their impact on long-term mortality. In the recommendations by Mensa et al. on severe infections caused by multidrug-resistant pathogens [11], the use of the CCI is suggested to inform antibiotic selection. Its inclusion in sepsis mortality prediction models remains under investigation [12,13,14].

Frailty is a clinical syndrome reflecting decreased functional reserve and diminished capacity to respond to stressors. It is associated with physiological alterations—such as immunosenescence, neuroendocrine dysregulation, and inflammatory changes—that predispose patients to infection and poorer clinical outcomes [15]. In the intensive care setting, frail patients account for 40% of patients over 75 years old [16] and have been shown to be at higher risk of organ dysfunction and in-hospital mortality [17,18], although its influence on outcomes outside the ICU and on clinical decision-making remains poorly defined.

Decision-making in septic patients is a complex process that requires a multidimensional approach, integrating not only biomarkers and organ dysfunction scores but also baseline health factors. This is particularly relevant given an aging population, where personalized clinical management is key to optimizing healthcare resources and improving risk stratification.

The primary objective of this study was to evaluate the influence of pre-existing health status (age, CCI, frailty and anemia) on in-hospital and 12-month mortality in patients with sepsis and septic shock, alongside initial clinical severity as measured by the SOFA score.

As a secondary objective, we aimed to examine how these baseline factors relate to ICU admission, thereby assessing whether patient characteristics affect triage patterns.

## 2. Materials and Methods

### 2.1. Study Design

An observational, analytical, retrospective study was conducted using the Sepsis Code database of Hospital Universitario La Princesa (Madrid, Spain).

### 2.2. Study Population and Inclusion Criteria

The study population comprised patients aged ≥ 18 years in whom the Sepsis Code was activated between 1 January 2016 and 1 July 2018 in any hospital area, including the emergency department; inpatient wards; medical ICU; and surgical ICU.

Each Sepsis Code alert was reviewed individually to select patients meeting the Sepsis-3 criteria for sepsis or septic shock. Patients with incomplete data for the study’s primary variables or whose alert activation did not correspond to a confirmed final diagnosis of sepsis were excluded. Of the 682 patients with a Sepsis Code alert, 547 met all inclusion criteria and had complete information for the key variables.

### 2.3. Sample Size Calculation

The sample size was sufficient to perform multivariable logistic regressions according to the events-per-variable rule (EPV ≥ 10), based on the number of independent variables analyzed and the observed in-hospital mortality rate (21%). Therefore, the study’s statistical power is considered adequate to detect clinically relevant associations.

### 2.4. Variables of the Study

The following variables were obtained through individual review of the medical records and were initially analyzed using bivariate logistic regression. Those with statistical significance or clinical relevance were included in a multivariate logistic regression.

#### 2.4.1. Independent Variables

• Sex.
• Age.
• Institutionalization, defined as residence in a long-term care facility before hospital admission.
• Primary infection source: respiratory; urinary; abdominal; unknown or other.
• Type of infection: community acquired or healthcare associated (nosocomial).
• Hospitalization within 30 days prior to the sepsis episode.
• Number of hospital admissions in the 12 months preceding the sepsis episode.
• Score obtained on the CCI (Figure S1) [10].
• Individual comorbidities as defined by the CCI.
• Frailty assessed using the Rockwood Clinical Frailty Scale (CFS) [19] only in patients above 65 years (Figure S2). Frailty was evaluated by a trained member of the research team who retrospectively reviewed the medical records of each patient. Whenever the scale score had been documented during admission by the clinical team, that value was used directly. In cases where no explicit score was recorded, the evaluator systematically examined clinical documentation from the six months prior to the sepsis episode—including previous hospital admissions, discharge summaries, progress notes, consultations, nursing comments, and recorded functional scales—to assign the frailty score that best reflected the patient’s baseline condition. Patients with a score ≥ 5 were considered frail. Cases in which the available documentation did not allow for a clear classification were excluded from the frailty analysis.
• Anemia on admission (hemoglobin < 12 g/dL in women and <13 g/dL in men, per World Health Organization criteria [20]).
• SOFA score within the first 24 h.

#### 2.4.2. Outcome Variables

• In-hospital mortality.
• Twelve-month mortality.
• Admission to medical or surgical ICU.

### 2.5. Statistical Analysis

Categorical variables are presented as counts and percentages and were compared using the chi-square (χ^2^) or Fisher’s exact test, as appropriate. Continuous variables are expressed as mean ± standard deviation; comparisons between two groups used Student’s *t*-test for normally distributed data or the Mann–Whitney U test for non-normal data. For comparisons among more than two groups, one-way ANOVA or the Kruskal–Wallis test was applied depending on data normality.

For the multivariate analysis, an explanatory model of mortality was constructed using logistic regression, which included all variables that were significant in the bivariate analysis. For each variable, Odds Ratios (ORs) with their 95% confidence intervals (CIs) were calculated. Statistical significance was set at *p* < 0.05.

All statistical analyses were conducted using SPSS version 19 (IBM Corp., Armonk, NY, USA) and Stata/SE version 13 (StataCorp, College Station, TX, USA).

## 3. Results

A total of 547 patients with sepsis or septic shock were ultimately included in the study. One hundred sixteen patients (21%) died during the hospitalization, and 184 (34%) died within 12 months. A total of 199 patients (36%) were admitted to the ICU. Most infections were community-acquired (82%), and the predominant infection foci were respiratory (36%), abdominal (29%), and genitourinary (22%). Baseline characteristics are shown in Table 1.

### 3.1. Age

The mean age was 73.7 ± 17 years, and 421 patients (77%) were over 65 years of age. The mean age was significantly higher among patients who died in hospital (73 vs. 78 years; *p* < 0.05) and among those who died by 12 months (72 vs. 77 years; *p* < 0.05).

Age > 65 years was associated with increased mortality in the bivariate analysis (Table 2). This association remained significant in the multivariate model only for 12-month mortality (Table 3).

### 3.2. Charlson Comorbidity Index

**Figure 1:**
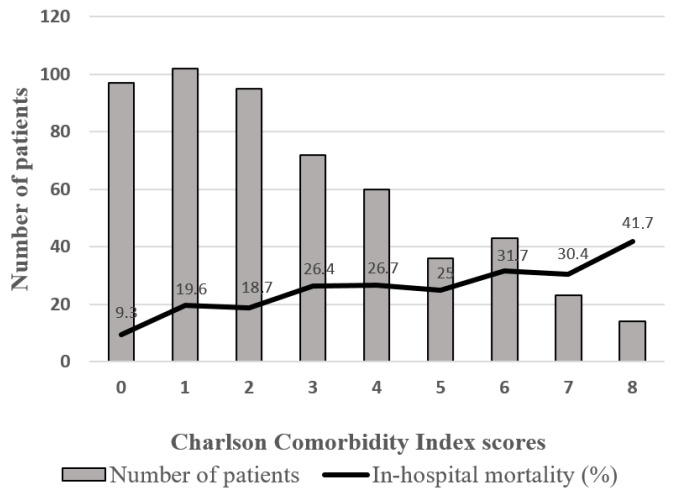
Number of patients by Charlson Comorbidity Index score and corresponding in-hospital mortality.

The mean CCI was 2.78 ± 1, with a median of 2 (range 0–13). Of the total cohort, 199 patients (36%) had a CCI < 2, while 348 (64%) had a CCI ≥ 2. Figure 1 depicts in-hospital mortality stratified by CCI score. The mean CCI was greater among those who died in hospital (2.58 vs. 3.42; *p* < 0.05) and those who died by 12 months (2.28 vs. 3.74; *p* < 0.05).

In the bivariate analysis, CCI ≥ 2 was significantly associated with both in-hospital and 12-month mortality (Table 2). However, in multivariate analysis no significant association was found between CCI ≥ 2 and mortality (Table 3).

### 3.3. SOFA

The mean initial SOFA score was 4.79 ± 2.5 (median 4; range 2–16). A total of 190 patients (35%) had SOFA < 4, while 357 (65%) had SOFA ≥ 4. Mean SOFA was significantly higher in non-survivors at discharge (4.43 vs. 6.17; *p* < 0.05) and in those who died within one year (4.31 vs. 5.74; *p* < 0.05).

SOFA ≥ 4 was associated with increased mortality in both bivariate and multivariate analyses (in-hospital mortality: OR 2.13, 95% CI 1.34–3.38; 12-month mortality: OR 2.05, 95% CI 1.35–3.10) (Table 3).

### 3.4. Frailty

Frailty, defined as CFS ≥ 5, was significantly associated with higher mortality in bivariate analysis both during hospitalization (OR 2.21, 95% CI 1.39–3.50) and at 12 months (OR 2.19, 95% CI 1.46–3.30) (Table 2).

This relationship persisted in multivariate models for both in-hospital mortality (OR 2.45, 95% CI 1.45–4.15) and 12-month mortality (OR 2.02, 95% CI 1.24–3.29) (Table 3).

### 3.5. Chronic Diseases

In the bivariate analysis, ischemic heart disease, severe liver disease, leukemia, and metastatic cancer were associated with in-hospital mortality (Table 2).

In the multivariate model, ischemic heart disease, severe liver disease, and metastatic cancer remained independently associated with increased in-hospital mortality (Table 3).

### 3.6. Anemia

Anemia on admission was significantly associated with both in-hospital and 12-month mortality in bivariate analysis (Table 2).

In multivariate analysis, anemia remained an independent predictor only of 12-month mortality (Table 3).

### 3.7. Infection Source

In bivariate analysis, respiratory sepsis was associated with higher mortality, whereas urinary sepsis was associated with lower mortality (Table 2).

In multivariate analysis, only urinary sepsis retained a protective association against in-hospital mortality; no other infection foci showed significant associations with mortality (Table 3).

### 3.8. ICU Admission

**Figure 2:**
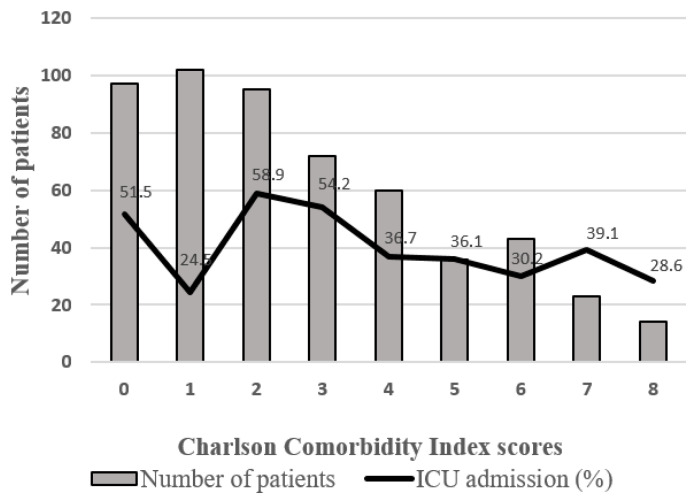
Number of patients by Charlson Comorbidity Index score and their Intensive Care Unit admission.

Thirty-six percent of patients (*n* = 199) were admitted to the medical or surgical ICU, with a mean ICU length of stay of 7.6 ± 2.8 days. ICU-admitted patients were younger (mean age 68.9 vs. 76.6 years; *p* < 0.05) and had a higher initial SOFA score (5.56 vs. 4.35; *p* < 0.05). Figure 2 shows the ICU admission rates stratified by CCI score.

In bivariate analysis, factors associated with increased ICU admission probability were SOFA ≥ 4, presence of solid malignant neoplasm, leukemia, nosocomial infection, and abdominal sepsis. Factors associated with decreased ICU admission included age ≥ 65 years, institutionalization, frailty, dementia, heart failure, and respiratory sepsis (Table 4).

In the multivariate analysis, SOFA ≥ 4, solid malignant neoplasm, nosocomial infection, and abdominal sepsis remained independently associated with higher ICU admission. Frailty, dementia, and heart failure were independently associated with lower ICU admission (Table 4).

## 4. Discussion

Individual patient characteristics influence the progression and outcomes of patients with sepsis. Frailty, the presence of specific comorbidities, age, and anemia were associated with higher in-hospital or 12-month mortality.

The SOFA score remains a robust tool for assessing severity and predicting outcomes in patients with sepsis. However, our results prove that its predictive capacity could be improved by adding the patient’s baseline condition, thus enabling a more personalized prognostic assessment.

Frailty has emerged as a highly relevant prognostic factor in elderly and septic patients. In our study, the presence of frailty was independently associated with increased in-hospital and 12-month mortality. This finding is consistent with previous studies conducted in ICUs, which have consistently demonstrated that frailty is associated with worse clinical outcomes, including higher mortality, functional decline, complications, and greater healthcare resource utilization [21,22,23]. However, the prognostic value of frailty is not limited to critical care settings. In the surgical context, several studies have shown that it is a strong predictor of postoperative mortality, complications, and prolonged hospital stay—often surpassing chronological age or comorbidity burden in predictive power [24,25,26]. Notably, the multicenter European POSE study, in which our hospital participated, found that preoperative frailty was significantly associated with increased 30-day mortality in patients over 80 years of age undergoing non-emergency procedures [27]. Specifically regarding intensive care, systematic reviews and meta-analyses have confirmed that frailty is a robust and independent prognostic marker associated with increased short- and long-term mortality and impaired recovery [28]. These findings support the need to systematically integrate frailty assessment into mortality prediction models and clinical decision-making.

Studies have confirmed that a higher CCI correlates with increased mortality in critically ill patients [29] and in those undergoing major surgery [30]. In sepsis specifically, the comorbidity burden measured by the CCI has been shown to be associated with worse outcomes, including increased mortality [12,31]. In our cohort, a higher CCI was associated with mortality in the univariate analysis, but did not retain statistical significance in the multivariate model. Although in our analysis the CCI did not behave as an overall independent predictor, comorbidities with significant prognostic impact were identified, such as ischemic heart disease, severe liver disease, and disseminated malignancy. These findings are consistent with previous studies [32] and suggest that these comorbidities should be taken into account when assessing prognosis and making therapeutic decisions in sepsis treatment. A septic patient with severe liver disease or metastatic cancer may have limited long-term prognosis, which could justify earlier goals-of-care discussions. By contrast, patients without such comorbidities might tolerate more aggressive interventions.

Another important factor is anemia. Although its role in sepsis has been less studied, our findings show that anemic patients have higher 12-month mortality. These results are particularly relevant given the high prevalence of anemia in our cohort (50%). Anemia may reflect chronic inflammation, malnutrition, or impaired oxygen transport, all of which can contribute to multi-organ failure and increased mortality in sepsis [33]. However, anemia did not influence ICU admission, suggesting that it is not yet considered in clinical decision-making despite its prognostic value. In a personalized approach, knowledge of pre-existing anemia might lead clinicians to optimize hemoglobin levels more aggressively in high-risk patients.

Regarding the infection source, our study shows that it affects both mortality and ICU admission decisions. Urinary tract infections were associated with lower in-hospital mortality, with no significant differences in ICU admission—possibly reflecting the lower severity of this sepsis type [34]. Abdominal sepsis did not show significant differences in mortality compared to other infection sources, but patients were more frequently admitted to the ICU. This may be due to its perceived severity and association with a higher risk of progression to septic shock or delayed source control [35]. Respiratory sepsis showed a trend toward higher mortality, although this was not sustained in the multivariate model. This aligns with previous studies indicating that respiratory infections are typically associated with greater clinical severity and the need for ventilatory support, both of which increase mortality risk [36]. Overall, these results reinforce the role of infection source in sepsis prognosis and management, emphasizing the importance of early identification for optimal decision-making and resource allocation.

The decision to admit a patient with sepsis to the ICU remains complex and is influenced by multiple clinical and non-clinical factors. In our study, patients with frailty, dementia, or heart failure were less likely to be admitted to the ICU, despite being at high risk of poor outcomes. This aligns with recent literature suggesting that, beyond acute severity, clinicians often integrate baseline characteristics such as age, frailty, comorbidity burden, and functional reserve into their decision-making process and can prioritize some patients based on individual clinical judgment—often in the absence of any evidence-based guidance [37,38,39,40]. While this individualized judgment may be clinically intuitive, it can lead to inequities in access to intensive care, particularly for older adults or those with chronic illnesses. Our data reinforce this concern: patients considered vulnerable were less frequently admitted to the ICU, yet presented with higher mortality, raising the question of whether some may have been denied potentially beneficial care. A personalized framework could help: structured protocols incorporating objective frailty and comorbidity assessments might ensure that ICU resources are allocated based on a transparent risk profile.

The long-term prognosis of patients who survive sepsis remains a relevant clinical concern. In our cohort, we observed a 12-month mortality rate of 34%, consistent with previous studies that describe excess late mortality among sepsis survivors [41,42]. Despite this, the underlying mechanisms behind this increased mortality are still not fully understood. Several studies suggest that, beyond the acute organ injury and the previous health status, there are pathogenic factors that may play a key role in the long-term vulnerability of these patients. Among them are accelerated cardiovascular pathology, a state of persistent inflammation, and immunosuppression [42,43]. Moreover, comorbidities such as cancer and heart diseases—which were associated with higher mortality in our study—are also known for their negative impact on long-term survival [44]. These findings reinforce the idea that 12-month mortality is not merely an extension of acute severity, but rather a reflection of accumulated vulnerability and limited physiological reserve.

In summary, these findings support the value of incorporating patient-specific variables into sepsis risk stratification and management. A personalized clinical pathway might stratify sepsis patients into risk subgroups based on the baseline health status. For example, high-risk patients (frail, with multiple comorbidities or anemia) might trigger an escalated management protocol, while low-risk patients could follow standard protocols if they improve quickly. In all cases, the intensity of interventions would be aligned with the patient’s profile.

Among the strengths of this study are the use of an extensive hospital database within a structured sepsis improvement program (Sepsis Code), and a detailed evaluation of each patient. Additionally, the multivariate analysis allowed us to identify independent predictors of mortality and assess their relative impact on the course of sepsis.

This study also has limitations. First, due to its retrospective design, the presence of bias and unmeasured confounding factors cannot be ruled out, as these may have influenced both mortality and clinical decisions made during hospitalization. Specifically, regarding the ICU admission of frail patients, no information was collected on decisions to limit therapeutic efforts. Therefore, it is not possible to determine whether the lower ICU admission rate among these patients reflects a care-related bias or an appropriate clinical judgment that led to limiting invasive interventions. Second, this is a single-center study, which limits the generalizability of the findings to other healthcare settings. Third, with regard to long-term outcomes, the causes of 12-month mortality or their potential relationship with the sepsis episode were not documented. This limits the ability to accurately interpret the long-term impact of the sepsis episode. Fourth, our cohort includes a clinically heterogeneous population. However, we believe that this variability reflects the real-world clinical setting of hospitalized patients with sepsis. Moreover, the use of multivariate models allowed for an adjustment of these factors and helped reduce the risk of confounding bias, thereby strengthening the validity of the findings. Finally, data collection based on electronic medical records carries the risk of human error, particularly in relation to the identification of frailty. This variable is not typically recorded in a systematic manner, which may have affected the accuracy of its assessment. To minimize the risk of information bias and improve the reliability of the assessment, continuous scale values were not used; instead, patients were grouped into two categories: frail (CFS ≥ 5) and non-frail (CFS < 5). Cases in which the available documentation did not allow for a clear classification were excluded from the frailty analysis.

## 5. Conclusions

Baseline health status influences the outcomes of patients with sepsis and septic shock. Frailty, specific comorbidities, age, anemia and source of infection should be incorporated into clinical decision-making and mortality prediction models. The integration of these variables could enhance tailored interventions and resource allocation that align with each patient’s profile, moving beyond “one-size-fits-all” guidelines toward individualized sepsis management.

## Figures and Tables

**Table 1:** Demographic, clinical characteristics and outcomes of patients with sepsis and septic shock.

Patient Characteristics	All Patients *n* = 547	ICU *n* = 199	Non ICU *n* = 348
Age, years ^a^	73.7 ± 17	68.9 ± 8.5	76 ± 2.1
<65, *n* (%)	126 (23)	62 (31)	64 (18)
≥65, *n* (%)	421 (77)	137 (69)	284 (82)
Male sex, *n* (%)	328 (60)	130 (65)	198 (57)
Place of residence, *n* (%)			
Non institutionalized	496 (91)	194 (97)	302 (87)
Institutionalized	46 (8)	5 (3)	41 (12)
Other	5 (1)	0 (0)	5 (1)
Rockwood Frailty Score, *n* (%)			
<5	226 (55)	104 (78)	122 (44)
≥5	184 (45)	29 (22)	155 (56)
CCI ^a^	2.78 ± 1.0	2.59 ± 1.4	2.88 ± 1.4
<2, *n* (%)	199 (36)	74 (37)	124 (36)
≥2, *n* (%)	348 (64)	125 (63)	224 (64)
Anemia, *n* (%)	271 (50)	102 (51)	169 (49)
Initial SOFA ^a^	4.8 ± 2.5	5.56 ± 2.8	4.35 ± 2.1
<4, *n* (%)	190 (35)	49 (25)	141 (41)
≥4, *n* (%)	357 (65)	150 (75)	207 (59)
Type of infection, *n* (%)			
Community-acquired	447 (82)	144 (72)	303 (88)
Nosocomial	96 (18)	55 (28)	41 (12)
Source of infection, *n* (%)			
Respiratory	196 (36)	51 (26)	145 (42)
Abdominal	160 (29)	82 (41)	78 (22)
Urinary	121 (22)	38 (19)	83 (24)
Unknown	32 (6)	12 (6)	20 (6)
Other	38 (7)	16 (8)	22 (6)
Contact with healthcare services			
Admission < 30 days, *n* (%)	81 (15)	25 (13)	56 (15)
Number admissions previous year ^a^	0.83 ± 0.5	0.77 ± 0	0.86 ± 0
0, *n* (%)	282 (55)	107 (58)	177 (54)
1–3, *n* (%)	213 (42)	72 (39)	141 (43)
≥4, *n* (%)	18 (3)	6 (3)	12 (4)
In-hospital mortality, *n* (%)	116 (21)	42 (21)	74 (21)
12-month mortality, *n* (%)	184 (34)	68 (34)	116 (34)

^a^ Mean ± standard deviation; *n*: frequency; ICU: Intensive Care Unit; CCI: Charlson Comorbidity Index; SOFA: Sequential Organ Failure Assessment.

**Table 2:** Bivariate analysis of mortality predictors in patients with sepsis and septic shock.

	In-Hospital Mortality			12-Month Mortality		
	OR	95% CI	*p* Value	OR	95% CI	*p* Value
Age ≥ 65	2.16	1.22–3.83	0.007	2.41	1.50–3.88	<0.001
CCI ≥ 2	2.06	1.35–3.13	0.001	3.05	2.10–4.42	<0.001
SOFA ≥ 4	2.47	1.62–3.78	<0.001	2.28	1.58–3.28	<0.001
Frailty (CFS ≥ 5)	2.21	1.39–3.50	0.001	2.19	1.46–3.30	<0.001
Ischemic heart disease	2.41	1.45–4.02	0.001	2.44	1.51–3.96	<0.001
Dementia			0.384	1.98	1.12–3.51	0.017
Severe liver disease	2.68	1.00–7.21	0.042			0.261
Leukemia	2.28	1.01–5.13	0.041	2.75	1.24–6.12	0.010
Disseminated oncologic disease	2.20	1.15–4.20	0.015	5.12	2.65–9.87	<0.001
Anemia	1.67	1.10–2.54	0.016	2.14	1.48–3.09	<0.001
Respiratory infection source	1.90	1.22–3.83	0.007	1.52	1.05–2.20	0.025
Urinary infection source	0.30	0.16–0.58	<0.001	0.51	0.32–0.82	0.004
Abdominal infection source	1.05	0.67–1.64	0.839	1.05	0.71–1.55	0.825

OR: Odds Ratio; CI: confidence interval; CCI: Charlson Comorbidity Index; SOFA: Sequential Organ Failure Assessment; CFS: Clinical Frailty Scale (Rockwood).

**Table 3:** Multivariate analysis of mortality predictors in patients with sepsis and septic shock.

	In-Hospital Mortality			12-Month Mortality		
	OR	95% CI	*p* Value	OR	95% CI	*p* Value
Age ≥ 65			0.18	1.98	1.09–3.59	0.025
CCI ≥ 2			0.57			0.33
SOFA ≥ 4	2.13	1.34–3.38	0.001	2.05	1.35–3.10	0.001
Frailty (CFS ≥ 5)	2.45	1.45–4.15	0.001	2.02	1.24–3.29	0.005
Ischemic heart disease	2.34	1.27–4.33	0.006	2.07	1.16–3.70	0.014
Dementia			0.96			0.10
Severe liver disease	3.62	1.09–12.10	0.036			0.25
Leukemia			0.14			0.06
Disseminated oncologic disease	3.14	1.43–6.90	0.004	6.15	2.84–13.3	<0.001
Anemia			0.10	1.85	1.21–2.84	0.005
Respiratory infection source			0.37			0.47
Urinary infection source	0.37	0.15–0.91	0.029			0.22
Abdominal infection source			0.85			0.22

OR: Odds Ratio; CI: confidence interval; CCI: Charlson Comorbidity Index; SOFA: Sequential Organ Failure Assessment; CFS: Clinical Frailty Scale (Rockwood).

**Table 4:** Predictors of admission in ICU.

	Bivariate Analysis			Multivariate Analysis		
	OR	95% CI	*p* Value	OR	95% CI	*p* Value
Age ≥ 65	0.5	0.33–0.75	0.001			0.53
SOFA ≥ 4	2.81	1.96–4.02	<0.001	3.69	2.40–5.66	<0.001
Frailty (CFS ≥ 5)	0.22	0.14–0.35	<0.001	0.20	0.09–0.41	<0.001
Institutionalization	0.18	0.07–0.45	<0.001			0.10
Dementia	0.06	0.01–0.24	<0.001	0.14	0.03–0.65	0.012
Leukemia	2.75	1.24–6.12	0.010			0.49
Heart failure	0.30	0.17–0.54	<0.001	0.45	0.23–0.89	0.022
Solid malignant neoplasm	2.81	1.70–4.63	<0.001	2.72	1.45–5.07	0.002
Nosocomial infection	2.82	1.80–4.43	<0.001	2.42	1.41–4.16	0.001
Respiratory infection source	0.48	0.33–0.71	<0.001			0.26
Abdominal infection source	2.43	1.66–3.54	<0.001	1.88	1.12–3.13	0.016

ICU: Intensive Care Unit; OR: Odds Ratio; CI: confidence interval; SOFA: Sequential Organ Failure Assessment; CFS: Clinical Frailty Scale.

## Data Availability

The anonymized dataset is available upon reasonable request from the corresponding author. The data is not public as it is protected.

## Supplementary Materials

The following supporting information can be downloaded at: https://www.mdpi.com/article/10.3390/jpm15090398/s1, Figure S1: Charlson Comorbidity Index (CCI), Figure S2: Rockwood Clinical Frailty Scale (CFS).

## Abbreviations

The following abbreviations are used in this manuscript:

SOFA	Sequential Organ Failure Assessment
ICU	Intensive Care Unit
CCI	Charlson Comorbidity Index
CFS	Clinical Frailty Score
OR	Odds Ratio
CI	Confidence Interval
