# Supplementary figures and images for: Frailty as an Independent Predictor of Mortality in Patients with Sepsis
Source: J Pers Med. 2025 Aug 26;15(9):398. doi: 10.3390/jpm15090398 (PMC12470791; doi:10.3390/jpm15090398)

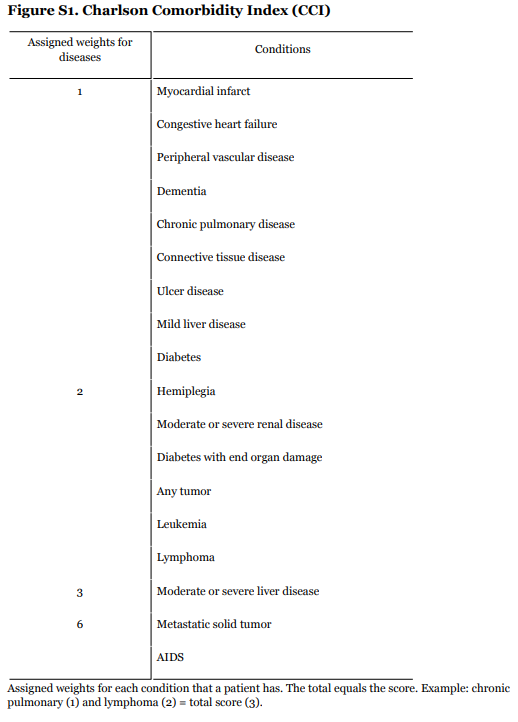

Supplement: Supplementary file 1 [file jpm-15-00398-s001.zip › jpm-3792470 (S1).png]

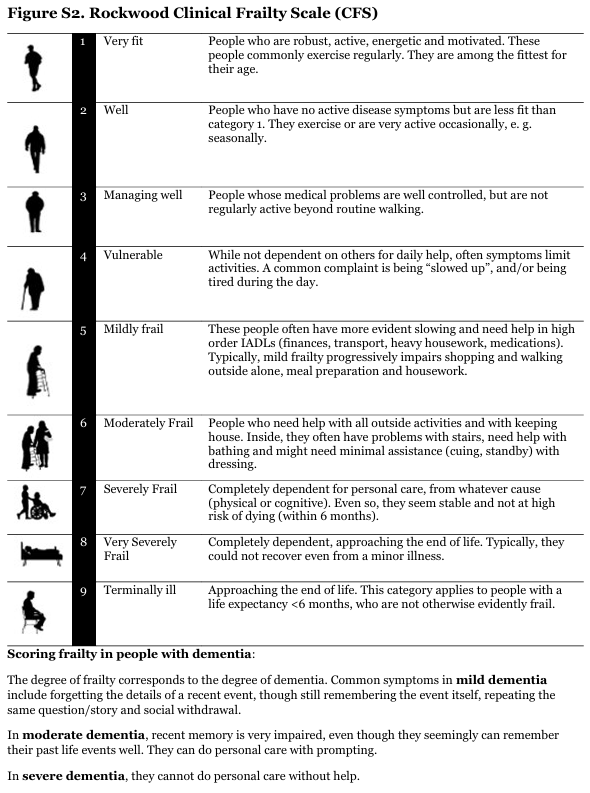

Supplement: Supplementary file 1 [file jpm-15-00398-s001.zip › jpm-3792470 (S2).png]
